# Glycine-Histidine-Lysine (GHK) Alleviates Neuronal Apoptosis Due to Intracerebral Hemorrhage via the miR-339-5p/VEGFA Pathway

**DOI:** 10.3389/fnins.2018.00644

**Published:** 2018-09-20

**Authors:** Heyu Zhang, Yanzhe Wang, Zhiyi He

**Affiliations:** Department of Neurology, The First Affiliated Hospital of China Medical University, Liaoning, China

**Keywords:** GHK, intracerebral hemorrhage, miR-339-5p, VEGFA, neuronal apoptosis

## Abstract

Glycine-histidine-lysine (GHK) is a human tripeptide that enhances wound healing, exerts neuroprotective effects against neurodegenerative disease, and improves tissue regeneration. This study examined whether GHK can alleviate injury due to intracerebral hemorrhage (ICH). Briefly, adult Wistar rats in GHK pretreatment groups were injected with GHK (1 or 10 mg/kg, i.p.) every 24 h for 3 days. Water content and intact neurons were detected in the rats 3 days after ICH, and the neurological deficit scores were examined in the rats at 4, 24, 72, and 168 h after ICH. Apoptosis was evaluated via caspase-3 immunohistochemistry, Nissl staining, and TUNEL assay. We also examined the effect of GHK on the expression of related proteins in SH-SY5Y cells via Western blotting. The expression of miR-339-5p was examined via real-time polymerase chain reaction analyses. GHK improved neurological deficits, reduced water content in the brain and inhibited neuronal apoptosis in ICH rats. It also prevented the apoptosis of SH-SY5Y cells with hemin treatment. Furthermore, GHK downregulated miR-339-5p expression, and overexpression of miR-339-5p partially reversed the anti-apoptotic effects of GHK in SH-SY5Y cells. Our findings suggest that the p38 MAPK pathway is involved in the GHK-induced downregulation of miR-339-5p, and that the miR-339-5p/VEGFA axis plays a role in preventing neuronal apoptosis following ICH injury. These findings indicate that GHK may represent a novel therapeutic strategy for ICH.

## Introduction

Ischemic and hemorrhagic stroke are among the leading causes of death and disability worldwide. Although it accounts for approximately 15% of all stroke cases, hemorrhagic stroke is associated with the highest mortality rates among the stroke subtypes, thus representing a major public health concern (Donnan et al., 2008; van Asch et al., 2010a). Primary damage is caused by the rupture of blood vessels in the brain, while secondary injuries include hematoma compression, perihematomal edema, neuronal death, and inflammation due to the effects of hemoglobin (Keep et al., 2012; Wang et al., 2014). Current treatment options for patients with intracerebral hemorrhage (ICH) are limited; therefore, timely and targeted treatment is critical.

Apoptosis refers to the process of programmed cell death that occurs in multicellular organisms. Neuronal apoptosis occurs following ICH due to hypoxia, inflammation, and hemoglobin toxicity. Recent studies have revealed that inhibition of neuronal apoptosis may improve ICH prognosis (Shi et al., 2012; Zille et al., 2017). However, the precise approach to maintain neuronal viability while preventing cell death due to vessel rupture and toxicity remains uncertain.

Currently available neuroprotective drugs exhibit limited efficacy (Qureshi et al., 2009); therefore, it is necessary to investigate potential therapeutic strategies that are both novel and safe. Naturally occurring substances may represent alternative options for ICH treatment. Glycine-histidine-lysine (GHK) is a human tripeptide that induces youth-like protein synthesis in aged human liver cells (Pickart et al., 1980). GHK generally exists in the plasma, urine, and saliva. Previous studies have suggested that GHK enhances wound healing and improves inflammation, emphysema-related lung destruction, and tissue regeneration (Campbell et al., 2012; Pickart et al., 2015). Additional studies have indicated that GHK may exert neuroprotective effects against neurodegenerative diseases (Pickart et al., 2012). However, the role of GHK and the mechanisms underlying its effects in patients with ICH remain to be elucidated. In the present study, we investigated the biological function of GHK in a rat model of ICH and hemin-induced SH-SY5Y cells.

## Materials and Methods

### Animal Preparation and Administration

All experimental protocols involving animals were performed according to National Institutes of Health Guide for the Care and Use of Laboratory Animals and ARRIVE (Animal Research: Reporting of *in vivo* Experiments) guidelines. Seventy-two male Wistar rats (250–280 g) were purchased from Liaoning Chang Sheng Biotechnology Co., Ltd. Animals were housed in an environmentally controlled room (22–25°C, 50% humidity) under a 12-h light–dark cycle and were provided *ad libitum* access to food and water. All procedures were conducted in accordance with the regulations of the animal protection laws of China and approved by the Animal Ethics Committee of China Medical University (2012-38-1).

Rats were randomly divided into three groups. (1) Control group (*n* = 24), rats underwent the collagenase VII-induced ICH surgical procedures and received vehicles intraperitoneally injected (i.p.) when the pretreatment groups were administered GHK. (2) GHK 1 mg/kg group (*n* = 24), rats in this group were injected with GHK (1 mg/kg, i.p.; every 24 h for 3 days) dissolved in saline prior to the administration of collagenase VII. (3) GHK 10 mg/kg group (*n* = 24), rats in this group were injected with GHK (10 mg/kg, i.p.; every 24 h for 3 days) dissolved in saline prior to the administration of collagenase VII. Twenty-four rats in each group were randomly divided into three groups by a researcher who was unaware of the neurological deficits in these rats. Eight rats were decapitated to obtain fresh brain tissue samples for water content. Eight rats were perfused with fixative for histological preparation and analysis of the brains. Eight rats were used for the neurological deficits scores until 168 h after ICH. The mortality of model preparation in this experiment is 16.67% in control group, 8.3% in GHK 1 mg/kg group and 4.16% in GHK 1 mg/kg group. All experimental data were collected and analyzed by an investigator who was unaware of the group in these rats.

### Model of Collagenase VII-Induced Intracerebral Hemorrhage

ICH was induced via stereotactic administration of 0.5 U bacterial collagenase type VII (Sigma, United States) in 2 μl saline, as previously described (Rosenberg et al., 1990).

### Analysis of Brain Water Content

Animals were anesthetized, and the brains were removed 72 h after ICH induction. The wet weight of each brain was immediately obtained using an electronic balance, following which the brains were dried at 100°C for 24 h to obtain the dry weight. Water content was calculated according to the following formula: [(wet weight-dry weight)/(wet weight) × 100 (%)] (Lee et al., 2008).

### Evaluation of Neurological Deficits

Neurological deficits were evaluated using a 5-point scale at 4, 24, 72, and 168 h after ICH induction, as previously described (Rosenberg et al., 1990).

### Immunohistochemistry and Nissl Staining

Immunohistochemistry experiments were performed using the UltraSensitiveTM SP (Mouse/Rabbit) IHC Kit (Mixim, Fu Zhou, China), in accordance with the manufacturer’s instructions. The brains were fixed with 4% paraformaldehyde for 24 h and dehydrated using 70–100% alcohol. Paraffin-embedded brains were cut into 5-μm sections. The slices were subjected to antigen retrieval under high-temperature and high-pressure conditions, following which they were treated with caspase-3 antibody (1:400), followed by endogenous peroxidase blockers. The slices were then blocked in serum overnight at 4°C. Subsequently, the slices were treated with secondary antibody (goat anti-rabbit), rinsed with PBS, stained with DAB, subjected to hematoxylin and eosin (H&E) staining, dehydrated, transparentized, and mounted prior to microscopic analysis. Nissl staining is used for detecting intact neurons. For Nissl staining, the tissues were treated with Cresyl Violet acetate for 15 min at 22–26°C, rinsed in PBS, dehydrated, transparentized, and mounted prior to microscopic analysis.

### TdT-Mediated dUTP Nick-End Labeling (TUNEL) Assay for Cell Apoptosis

TUNEL assay were performed using the *in situ* Cell Death Detection Kit (Roche, Mannheim Germany.) Brain sections were stained according to the manufacturer’s instruction. In brief, slices washed with PBS three times, fixed with 4% paraformaldehyde for 30 min, incubated in 0.5% Triton X-100 for 5 min, then incubated in TUNEL reaction mixture for 60 min at 37°C. Nuclei were stained by DAPI (1:2000) for 2 min. The results were observed using a fluorescent microscope.

### Cell Culture and *in vitro* Model of Hemorrhagic Toxicity

Human-derived SH-SY5Y cells (Chinese Academy of Sciences, Shanghai) are commonly used as *in vitro* models of neuronal function and differentiation. SH-SY5Y cells were propagated in DMEM/F12 (Gibco, UA) supplemented with 10% fetal bovine serum (Gibco, Australia), following which they were incubated at 100% humidity in an environment containing 5% CO_2_. To imitate neuronal injury after hemorrhagic stroke, an *in vitro* model was constructed by applying 100 μM hemin to SH-SY5Y cells (control group).

### Synthetic RNA Oligonucleotides and Transfection

We obtained microRNA-339-5p (miRNA-339-5p) mimics, miRNA-339-5p inhibitors, and a nonsense sequence, as the miRNA negative control (NC), from GenePharma (Shanghai, China). SH-SY5Y cells were transfected with Lipofectamine 2000 (Invitrogen, Carlsbad, CA, United States), in accordance with the manufacturer’s instructions.

### RNA Extraction and Real-Time PCR

Total RNA was extracted from cells using TRIzol reagent, in accordance with the manufacturer’s protocol (Invitrogen, Carlsbad, CA, United States). Reverse transcription of microRNA (miRNA) was performed using the hairpin-it miRNAs qPCR Quantitation Kit (GenePharma, Shanghai, China), in accordance with the manufacturer’s protocol. The PrimeScript RT Reagent Kit (TaKaRa Bio, Dalian, China) was used for mRNA reverse transcription-PCR. Real-time PCR was performed using the QuantiTect SYBR Green PCR Kit (TaKaRa Bio, Dalian, China) and an ABI 7500 system (ThermoFisher, Carlsbad, CA, United States). The relative miRNA expression of each gene was normalized to the expression of U6 RNA, while the relative mRNA expression of each gene was normalized to the expression of β-actin mRNA. The primers were synthesized by Sangon Biotech (Shanghai, Co., Ltd.). All primer sequences are listed in **Supplementary Table S1**.

### Western Blot Analysis

SH-SY5Y cells were lysed in RIPA (Beyotime, Beijing, China) containing inhibitor cocktail (Roche, Germany) for 30 min, following which the lysates were centrifuged (12,000 × *g*) at 4°C for 15 min. Protein concentration was determined using a BCA Protein Kit (Thermo, Rockford, IL, United States). Cell lysates were separated via 4–12% sodium dodecyl sulfate polyacrylamide gel electrophoresis (SDS-PAGE), following which the proteins were transferred to polyvinylidene difluoride (PVDF) membranes. The membranes were then incubated in 1% bovine serum albumin (BSA) solution with the following primary antibodies overnight at 4°C: anti-vascular endothelial growth factor A (anti-VEGFA; Abcam, ab46157), anti-p38 (Cell Signaling Technology, 9926), anti-phospho-p38 (Cell Signaling Technology, 9910), anti-general transcription factor II-I (anti-GTF2I; Abcam, ab134133), anti-membrane-associated guanylate kinase (anti-MAGI2; Abcam, ab97343), and anti-β actin (Abcam, ab8227). The membranes were then incubated with secondary antibody for 1 h at room temperature.

### Analysis of Cellular Apoptosis

Flow cytometric analysis of apoptosis was performed based on the quantitative detection of phosphatidylserine on the cell surface using an Annexin V/FITC and PI Apoptosis Detection Kit (BD, CA, United States). Following transfection with miR-339-5p mimics and NCs for 24 h, respectively, the cells were treated with or without GHK. Cells were washed twice in cold phosphate buffered saline (PBS) and suspended in 1X binding buffer. The cell suspension (100 μl) was incubated with Annexin V-FITC (5 μl) and propidium iodide (PI) (5 μl) for 15 min at room temperature. Binding buffer (400 μl) was then added to the cell suspension, and the samples were analyzed within 1 h using a flow cytometer (BD, CA, United States).

### Cell Counting Kit-8 (CCK-8) Assay for Cell Viability

Cell viability was determined using CCK-8 solution (Beyotime, Beijing, China), in accordance with the manufacturer’s instructions. Approximately 5 × 10^3^ cells were seeded in each well of a 96-well plate for 6 h, followed by incubation with hemin for 18 h. A total of 10 μl of CCK-8 solution was added to each well, and the cells were incubated for 1 h at 37°C. The absorbance of each well was quantified at 450 nm using an automated ELISA reader (SpectraMax^®^ M5, Molecular Devices, United States). Cell viability was calculated as follows: (A450 of transfected wells/A450 of control wells) × 100%.

### Lactate Dehydrogenase (LDH) Assay for Cell Injury

Cytotoxicity was determined using an LDH Assay Kit (KeyGen, Nanjing, China), in accordance with the manufacturer’s instructions. Following treatment with hemin for 18 h, the supernatant was collected and transferred to 96-well plates to measure LDH release. The absorbance of each sample was quantified at 450 nm using an automated ELISA reader (SpectraMax^®^ M5, Molecular Devices, United States).

### Dual Luciferase Assay

SH-SY5Y cells (1 × 10^4^ cells/well) were seeded onto 24-well plates and cultured overnight, following which they were transfected with wild-type and mutant *VEGFA* promoter-luciferase plasmids (0.1 μg pmirGLO-wt-VEGFA or pmirGLO-mt-VEGFA plasmid per well) using Lipofectamine 2000 (Invitrogen, CA, United States). The cells were co-transfected with either 0.4 μg miR-339-5p mimics or 0.4 μg miR-339-5p NCs (GenePharma, Shanghai, China). Transfection efficiency was standardized based on that of TK activity. Luciferase activity was quantified using a dual-luciferase assay system (Promega, E1910).

### Statistical Analysis

All data were analyzed using SPSS 23.0 software (IBM Corp., Armonk, NY, United States) and GraphPad Prism 5.0 (GraphPad Software, Inc., La Jolla, CA, United States). The level of statistical significance was set at *p* < 0.05. Each assay was repeated independently at least three times, and the measurement data are expressed as the mean ± standard deviation. Statistical significance between two groups was assessed using a two-tailed Student’s *t*-test (α = 0.05). One-way analysis of variance (ANOVA) followed by Dunnett’s or SNK test was performed for multiple comparisons. A prior power analysis was performed with the G^∗^Power 3.1.9.2 software at 5% significance level to determine the sample size of animals per group. We got a power greater than 0.9.

## Results

### GHK Alleviated Neuronal Apoptosis and Neurological Deficits Following ICH

**FIGURE 1:**
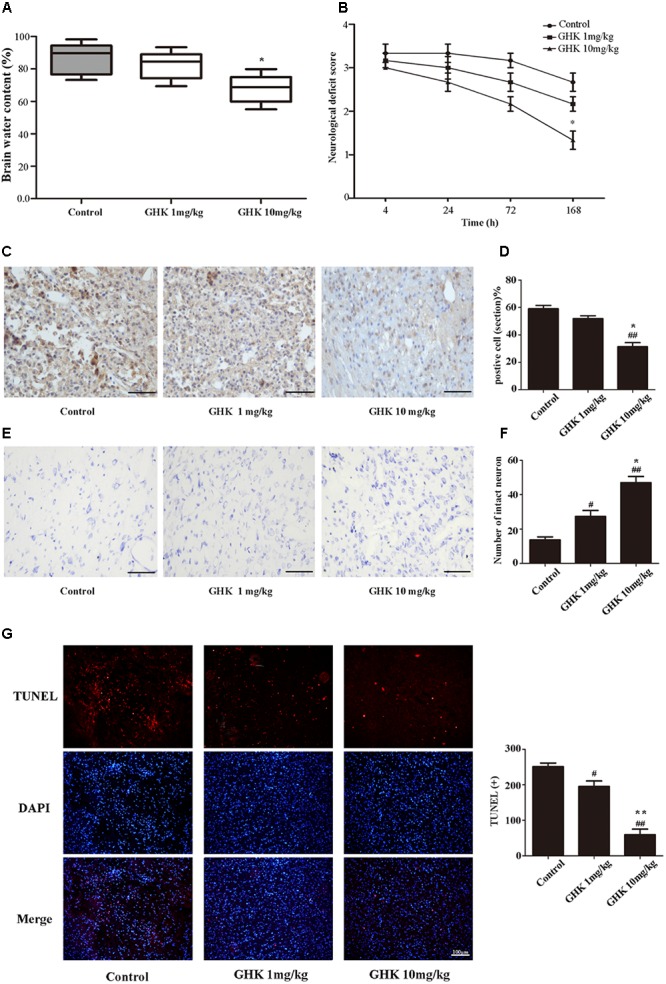
Glycine-histidine-lysine (GHK) alleviated neuronal apoptosis and neurological deficits following intracerebral hemorrhage (ICH). **(A)** GHK delivery via the intraperitoneal route decreases water content in the brain 72 h after ICH. **(B)** Analyses of neurological deficits revealed that treatment with both 1 mg/kg and 10 mg/kg GHK facilitates neurological recovery at 7 days after ICH. **(C,D)** Immunohistochemistry analysis of caspase-3 expression. Paraffin slices (5 μm) from the control and GHK groups were stained with caspase-3. (scale bar = 100 μm). **(E,F)** Nissl staining *in vivo*. Paraffin slices (5 μm) from the control and GHK groups were stained with Cresyl Violet acetate. (scale bar = 100 μm). **G**. TUNEL staining *in vivo*. Frozen slices (10 μm) were stained with TUNEL kit. (Data are represented as the mean ± SD, *n* = 8. ^#^, *p* < 0.05 vs. control, ^##^, *p* < 0.01 vs. control, ^∗^, *p* < 0.05 vs. GHK 1 mg/kg, and ^∗∗^, *p* < 0.01 vs. GHK 1 mg/kg).

To investigate the effect of GHK on ICH injury, we isolated the rat brain 3 days after administration of collagenase VII. We measured water content, caspase-3 expression, and the number of surviving neurons in the brain. Neurological deficits were detected 4, 24, 72, and 168 h after the administration of collagenase VII. Our results indicated that treatment with GHK significantly decreased water content (**Figure 1A**) and improved neurological deficit scores when compared to the control group (**Figure 1B**). In addition, GHK reduced the expression of caspase-3 in the brain (**Figures 1C,D**), and significantly increased the number of surviving neurons (**Figures 1E,F**). Moreover, TUNEL assays revealed that GHK significantly decreased cell apoptosis in ICH rats (**Figure 1G**).

### GHK Alleviated Hemin-Induced Apoptosis of SH-SY5Y Cells

**FIGURE 2:**
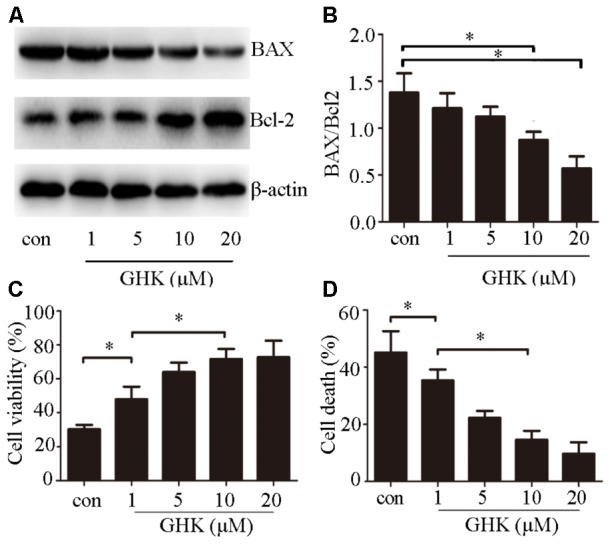
Glycine-histidine-lysine (GHK) alleviates hemin-induced apoptosis of SH-SY5Y cells. **(A,B)** Bax and Bcl-2 expression in GHK-treated SH-SY5Y cells. GHK treatment decreases the rate of Bax/Bcl-2 expression following hemin treatment. **(C)** GHK treatment increases the survival of SH-SY5Y cells following hemin-induced injury in a concentration-dependent manner. **(D)** GHK treatment decreases LDH release in a concentration-dependent manner. (Data are represented as the mean ± SD, *n* = 3, and ^∗^*p* < 0.05.)

To investigate the effects of GHK on ICH injury, we estimated the expression of Bax/Bcl-2 (**Figures 2A,B**). We further examined cell viability using CCK-8 (**Figure 2C**) and cell death rates using an LDH assay (**Figure 2D**) 24 h after treatment with GHK. Our findings indicated that GHK significantly alleviated hemin-induced apoptosis of SH-SY5Y cells when compared to the control group.

### GHK Downregulated miR-339-5p Expression in Hemin-Treated SH-SY5Y Cells, While Overexpression of miR-339-5p Increased Apoptosis of SH-SY5Y Cells

**FIGURE 3:**
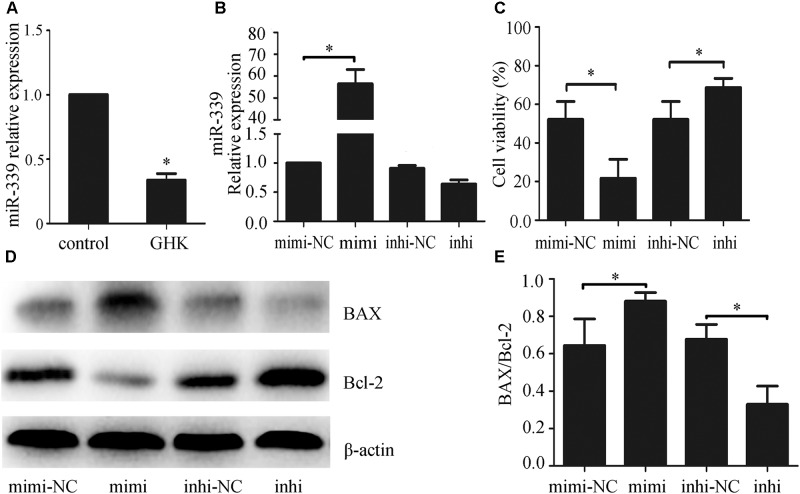
Glycine-histidine-lysine (GHK) treatment downregulated miR-339-5p expression in hemin-treated SH-SY5Y cells, while miR-339-5p overexpression increased the apoptosis of SH-SY5Y cells. **(A)** Decreased expression of miR-339-5p in SH-SY5Y cells following GHK treatment. SH-SY5Y cells were incubated with GHK (10 μM), and miR-339-5p levels were measured after 48 h. **(B)** Transfection efficiency of miR-339-5p. **(C)** miR-339-5p aggravates SH-SY5Y cell injury. **(D,E)** Bax and Bcl-2 expression after transfection of mimics, inhibitors, and negative controls (NCs). Transfection of miR-339-5p mimics upregulated Bax/Bcl-2 expression, while transfection of inhibitors has the opposite effect. (Data are presented as the mean ± SD, *n* = 3 and ^∗^*p* < 0.05.)

Previous studies have revealed that miR-339-5p may be involved in producing the effects of stroke (Altintas et al., 2016), and that GHK may alleviate the development of oxidative stress and anoxia (Canapp et al., 2003), which represents the main cause of stroke. However, no studies have explored the mechanism via which GHK acts on miR-339-5p. To investigate the potential role of miR-339-5p in hemin-induced neural cell injury, we examined the expression of miR-339-5p in hemin-treated SH-SY5Y cells via qPCR. Our results indicated that miR-339-5p expression was significantly lower in SH-SY5Y cells subjected to GHK treatment than in those of the control group (**Figure 3A**), suggesting an association between GHK and miR-339-5p. To further explore the role of miR-339-5p in hemin-treated SH-SY5Y cells, mimics or inhibitors of miR-339-5p were transfected into SH-SY5Y cells (**Figure 3B**). Transfection of miR-339-5p inhibitors inhibited the apoptosis of SH-SY5Y cells and promoted cell viability, whereas enhancing the expression of miR-339-5p produced the opposite effects (**Figures 3C–E**). These results suggest that downregulation of miR-339-5p promotes SH-SY5Y cell survival following ICH.

### *VEGFA* was a Functional Target Gene of miR-339-5p

**FIGURE 4:**
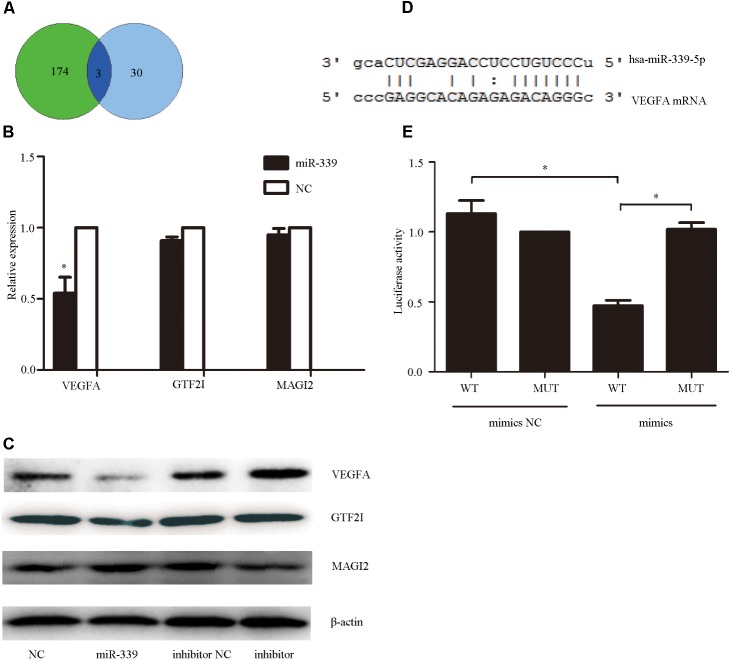
Vascular endothelial growth factor A (VEGFA), the functional target gene of miR-339-5p. **(A)**. To determine the mechanism via which miR-339-5p acts on SH-SY5Y cells, we predicted the possible target genes of miR-339-5p. The results were examined in conjunction with the effects of GHK treatment to identify intersectional effects on neuronal gene expression for three genes. **(B,C)** Levels of mRNA and protein expression for general transcription factor II-I (GTF2I), membrane-associated guanylate kinase (MAGI2), and *VEGFA* in GHK-treated SH-SY5Y cells, compared with control values. **(D)** Binding sites for miR-339-5p and *VEGFA*. **(E)** Levels of luciferase activity in SH-SY5Y cells co-transfected with miR-339-5p and *VEGFA*. (Data are represented as the mean ± SD, *n* = 3 and ^∗^*p* < 0.05.)

To determine the mechanism underlying miR-339-5p actions on SH-SY5Y cells, we utilized miRwalk^1^ to predict the possible target genes of miR-339-5p. The results were examined in conjunction with the effects of GHK treatment to identify intersectional effects on neuronal gene expression.(Pickart et al., 2017) Intersections were observed for *GTF2I, MAGI2*, and *VEGFA* (**Figure 4A**). Among them, *VEGFA*–an angiogenesis and neurotrophic factor–was selected for use in subsequent experiments (**Figure 4B**). To confirm whether *VEGFA* expression is regulated by miR-339-5p, we assessed VEGFA protein levels in SH-SY5Y cells transfected with miR-339-5p mimics, inhibitors, or NCs. Overexpression of miR-339-5p significantly reduced VEGFA protein levels in SH-SY5Y cells, while transfected miR-339-5p inhibitors produced the opposite effects (**Figure 4C**). We then cloned the 3′-UTR fragment of *VEGFA* mRNA containing the putative miR-339-5p binding sites, as well as the mutant 3′-UTR fragment lying upstream of the luciferase coding sequence (**Figure 4D**). The level of luciferase activity was reduced in the cells co-transfected with miR-339-5p mimics and *VEGFA* mRNA 3′-UTR fragments. However, no significant difference was observed in those transfected with the miR-339-5p mimic and mutant 3′-UTR fragment, indicating that miR-339-5p may target *VEGFA* mRNA to negatively regulate the expression of VEGFA (**Figure 4E**). These results suggest that *VEGFA* is a direct target of miR-339-5p, in turn suggesting that miR-339-5p produces its effects on ICH by targeting *VEGFA*.

### GHK Decreases miR-339-5p Levels via the p38 Pathway, While miR-339-5p Overexpression Prevents GHK-Induced Increases in *VEGFA* Expression in Hemin-Treated SH-SY5Y Cells

**FIGURE 5:**
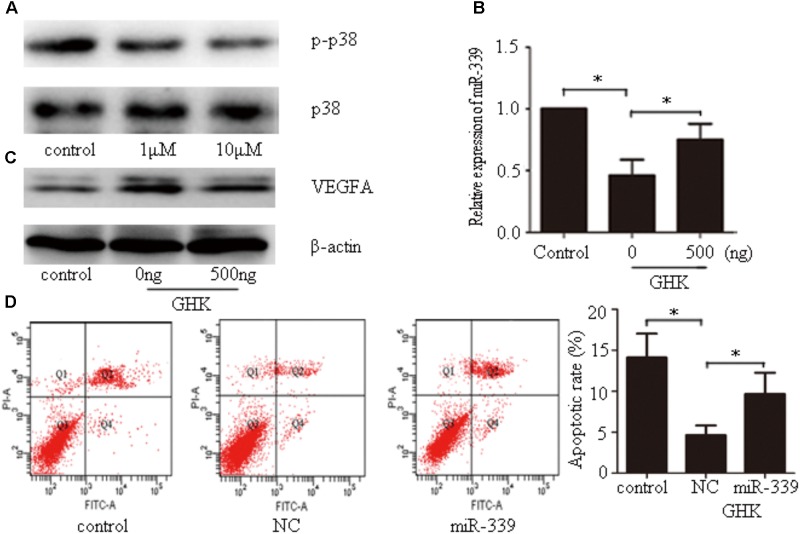
Glycine-histidine-lysine (GHK) decreases miR-339-5p levels via the p38 pathway, while miR-339-5p overexpression prevents GHK-induced increases in *VEGFA* expression in SH-SY5Y cells treated with hemin. **(A)** Expression of phosphorylated p38 in GHK-treated SH-SY5Y cells. SH-SY5Y cells were incubated with 1 μM or 10 μM GHK. **(B)** Expression of miR-339-5p in SH-SY5Y cells stimulated with GHK and p38 activator. Cells were treated with GHK (10 μM) and different concentrations of the p38 activator dehydrocorydaline. Levels of miR-339-5p were detected 24 h after treatment. **(C)** Expression of *VEGFA* in SH-SY5Y cells stimulated with GHK and p38 activator. Cells were treated with GHK (10 μM) and different concentrations of the p38 activator dehydrocorydaline. Levels of miR-339-5p were detected 48 h after treatment. **(D)** The apoptotic rate of SH-SY5Y cells. SH-SY5Y cells were stimulated with GHK and transfected with miR-339-5p. (Data are shown as the mean ± SD, *n* = 3 and ^∗^*p* < 0.05.)

Our findings indicated that GHK significantly downregulates the phosphorylation of p38 (**Figure 5A**). To investigate the mechanism by which GHK decreased the expression of miR-339-5p, SH-SY5Y cells that had been simultaneously stimulated with GHK and hemin were subsequently treated with different concentrations of the p38 activator dehydrocorydaline. Our results indicated that dehydrocorydaline reversed the downregulation of miR-339-5p induced by GHK (**Figure 5B**). Our findings also indicated that GHK treatment alleviated SH-SY5Y injury via miR-339-5p/VEGFA through the p38 pathway. To further investigate the relationship between GHK and miR-339-5p, cells were transfected with miR-339-5p mimics 24 h prior to GHK treatment. We observed decreased *VEGFA* expression and increased neuronal apoptosis in SH-SY5Y cells transfected with miR-339-5p mimics when compared with NCs (**Figures 5C,D**).

## Discussion

Plasma levels of the human tripeptide GHK tend to decrease with age, and have been associated with wound healing in several previous studies (Pickart, 2008; Pickart et al., 2012, 2015). GHK is non-toxic, natural, and exerts its effects at very low concentrations (Park et al., 2016). Recent studies have demonstrated that GHK is associated with tissue regeneration, anti-inflammatory and anti-oxidant effects, and the synthesis of neurotrophic factors (Jose et al., 2014; Pickart et al., 2014). As poor outcomes following ICH result from neuronal injury, inhibiting neuronal apoptosis in the early stage is critical for improving functional neurological recovery (van Asch et al., 2010b). Previous studies have reported that GHK reduces oxidative injury by regulating iron levels (Miller et al., 1990; Canapp et al., 2003). Thus, we examined the effect of GHK on neuronal apoptosis following ICH both *in vivo* and *in vitro*. To the best of our knowledge, our findings are the first to indicate that ICH rats pretreated with GHK exhibit significant decreases neurological functional deficits and the apoptosis of neural cells compared with the control rats. In addition, SH-SY5Y cells treated with GHK exhibited significant increases in cell viability as well as decreases in the level of LDH release caused by hemin-induced injury. These results suggest that GHK plays an important role in neuronal apoptosis after ICH.

As an important form of non-coding RNAs, miRNAs contain 21–23 nucleotides that act to negatively regulate the expression of target genes, mainly by binding to the 3′-UTR of their mRNA (Carthew and Sontheimer, 2009). Located at 7p22.3, miR-339-5p is known to inhibit tumor cell proliferation, promote tumor cell apoptosis (Yan et al., 2016), cleave β-site amyloid precursor protein, regulate genes associated with neurodegenerative diseases, and inhibit the development of medulloblastoma (Genovesi et al., 2012; Vallelunga et al., 2014; Liu et al., 2015). In the present study, we observed that expression of miR-339-5p was downregulated in SH-SY5Y cells following GHK treatment, compared with control models of ICH. Further analyses suggested that downregulation of miR-339-5p decreased neuronal apoptosis and promoted cell viability, while overexpression of miR-339-5p produced the opposite effects. These results suggest that regulation of miR-339-5p may attenuate the effects of ICH injury in SH-SY5Y cells.

Our results further indicated that miR-339-5p negatively regulates the expression of *VEGFA* by binding to the 3′-UTR. VEGFA has been identified as an effective and selective endothelial cell mitogen implicated in vascularization and angiogenesis (Yang et al., 2014). In addition, previous studies have indicated that transgenic mice overexpressing *VEGF* and rats subjected to virus-mediated *VEGF* gene transfer exhibit increases in performance on associative and spatial memory tasks (Cao et al., 2004; Plaschke et al., 2008). Additional studies have demonstrated that VEGFA promotes neuronal survival and neurite outgrowth in primary cortical neurons and dorsal root ganglia (Sondell et al., 1999; Rosenstein et al., 2003). Furthermore, VEGFA is an independent neurotrophic factor involved in nerve development and recovery that may exert neuroprotective effects by stabilizing or upregulating the expression of MAP2 and some other proteins (Rosenstein et al., 2003; Rosenstein and Krum, 2004; Pickart et al., 2017). Since VEGFA is too large to pass through the blood-brain barrier, we investigated the potential regulatory effect of GHK on *VEGFA* in ICH. Our findings indicated that GHK significantly upregulated levels of *VEGFA* expression, which in turn downregulated LDH release, decreased neuronal apoptosis, and promoted cell viability. In addition, the upregulation of *VEGFA* was reversed by overexpression of miR-339-5p, suggesting that miR-339-5p exerts its effects on ICH-induced injury by targeting *VEGFA*.

Previous studies have identified several targets regulated by GHK. Park et al. (2016) revealed that GHK significantly inhibits the p38 MAPK pathway in acute lung injury, while Pickart et al. (2017) demonstrated that GHK promotes the expression of neuronal VEGFA in those with neurodegenerative diseases. An additional study reported that VEGF exerts protective effects against caspase-mediated cell death, and that this process is negatively regulated by the p38 MAPK pathway (Gomes and Rockwell, 2008). Lyu et al. (2018) further observed that inhibiting the p38 MAPK pathway upregulates VEGF expression–a process that plays a role in the protective effects of vitexin against sevoflurane-induced neuronal apoptosis in newborn rats. In accordance with these previous studies, we observed that GHK significantly inhibited activation of the p38 MAPK pathway and decreased the expression of miR-339-5p. Moreover, activating the p38 MAPK pathway decreased the neuroprotective effects of GHK, which reversed the downregulation of miR-339-5p and upregulation of VEGF. These findings suggest that the p38 MAPK pathway is involved in the neuroprotective effect of GHK and plays a role in the negative regulation of neuronal VEGF in neural cells on ICH rats. However, further studies are required to more fully elucidate the relationship between the p38 MAPK pathway and VEGF following GHK treatment.

Accumulating evidence showed the possible methods of therapeutic use of GHK. Hostynek et al. (2010) demonstrated that GHK could rapidly pass through skin’s stratum corneum and reach therapeutically effective amounts and the use of encapsulated liposomal GHK would allow its oral administration at relatively high dosages (Pickart et al., 2017). These findings support the possibility and safety of using GHK orally to treat ICH.

The present study possesses some limitations of note. We investigated the role of GHK on neurons only, rather than on both neurons and neuroglia. Hence, the effect of GHK on ICH should be further explored.

In conclusion, our findings demonstrated that GHK alleviated neurological deficits in a rat model of ICH and decreased the apoptosis of SH-SY5Y cells following *in vitro* hemin treatment. In addition, we observed that GHK treatment downregulated miR-339-5p expression and decreased apoptosis of SH-SY5Y cells, likely via upregulation of *VEGFA*. Furthermore, our findings suggested that inhibition of the p38 MAPK pathway is involved in the GHK-induced downregulation of mir-339-5p, and that the miR-339-5p/VEGFA axis plays a role in neuronal anti-apoptosis following ICH injury. Ultimately, these findings indicate that GHK may be a novel therapeutic strategy for ICH.

## Author Contributions

ZH designed the experiments. HZ and YW performed the experiments and analyzed data. HZ wrote the manuscript.

## Conflict of Interest Statement

The authors declare that the research was conducted in the absence of any commercial or financial relationships that could be construed as a potential conflict of interest.

## Footnotes

1 http://zmf.umm.uni-heidelberg.de/apps/zmf/mirwalk2/index.html
