# Supplementary material for: Glycine-Histidine-Lysine (GHK) Alleviates Neuronal Apoptosis Due to Intracerebral Hemorrhage via the miR-339-5p/VEGFA Pathway
Source: Front Neurosci. 2018 Sep 20;12:644. doi: 10.3389/fnins.2018.00644 (PMC6158323; doi:10.3389/fnins.2018.00644)
Supplement: Supplementary file 1 [file Table_1.DOCX]

Table 1

|  | Forward | Reverse |
| --- | --- | --- |
| miR-339 | 5′-TGCCAGTTAGTAGCCCAGAAGCAA-3′ | 5′-TGATGTGCCAGGGAAGAAAGCCTA-3′ |
| U6 | 5′-ATTGGAACGATACAGAGAAGATT-3′ | 5′-GGAACGCTTCACGAATTTG-3′ |
| VEGFA | 5′-CTGAGGAGTCCAACATCACCATGC-3′ | 5′-CGCCTCGGCTTGTCACATCTG-3′ |
| GTF2I | 5′-AAGCTGTGAAGGTGCCGTTCG-3’ | 5′-GTGCTCTCCTTAATCGCCGTCTC-3’ |
| MAGI2 | 5′-CAGCACCACCTCAACCACTTCAG-3’ | 5′-ATGGAGGCTGTCGGATGTCTGG-3’ |
| β-actin | 5′-TGTCACCAACTGGGACGATA-3′ | 5′-GGGGTGTTGAAGGTCTCAAA-3′ |
